# Population mobility induced phase separation in SIS epidemic and social dynamics

**DOI:** 10.1038/s41598-020-64183-1

**Published:** 2020-05-06

**Authors:** Nathan Harding, Richard E. Spinney, Mikhail Prokopenko

**Affiliations:** 1 Centre for Complex Systems, Faculty of Engineering, University of Sydney, Sydney, NSW 2006 Australia; 2 Marie Bashir Institute for Infectious Diseases and Biosecurity, University of Sydney, Westmead, NSW 2145 Australia

**Keywords:** Computational models, Infectious diseases

## Abstract

Understanding the impact of behavior dependent mobility in the spread of epidemics and social disorders is an outstanding problem in computational epidemiology. We present a modelling approach for the study of mobility that adapts dynamically according to individual state, epidemic/social-contagion state and network topology in accordance with limited data and/or common behavioral models. We demonstrate that even for simple compartmental network processes, our approach leads to complex spatial patterns of infection in the endemic state dependent on individual behavior. Specifically, we characterize the resulting phenomena in terms of phase separation, highlighting phase transitions between distinct spatial states and determining the systems’ phase diagram. The existence of such phases implies that small changes in the populations’ perceptions could lead to drastic changes in the spatial extent and morphology of the epidemic/social phenomena.

## Introduction

Understanding and modelling population mobility during epidemics has been highlighted as a key area of importance for epidemiology^1–3^, especially in cases when the epidemics occur over a prolonged time period. Recent Ebola virus disease (EVD) outbreaks in western Africa during 2014–2016, and the even more recent outbreaks in heavily populated regions in the Democratic Republic of the Congo (DRC) emphasized the complexity of the disease spread in volatile and diverse areas. The efforts to contain the virus have been hindered by the high rates of population movement from the affected areas to other areas of the DRC and across borders to neighboring countries, creating complex paths and patterns of the infection spread.

In cases like these, high rates of mobility characterize both infected and uninfected populations due to the general lack of security, long-held mistrust of authority and wide-spread community distrust. Hence, the nature and dynamics of spatial spread of infectious diseases^4,5^ must be investigated and modelled explicitly, ideally coupled with more established network-based^6–17^ and/or agent-based methods of computational epidemiology^18–21^. However, there are several challenges on this path, making the development of refined epidemic models problematic.

One significant obstacle is the lack of high-resolution data, both in terms of contact rates and transmission vectors, specified within and across suitable social mixing contexts. Despite successful attempts to utilize proxy data, such as mobile phone usage data, in retrospective modelling of the population mobility in highly volatile regions during the Ebola epidemic in Sierra Leone in 2015^22^, there are several outstanding questions about spatial epidemic expansion, related to (i) the role of density-dependent transmission, especially in situations where mobility restrictions may increase or decrease close congregation, or in cases with highly local super-spreading events (e.g., burials), and (ii) the role of long-distance travel.

Another challenge for spatial modelling is the heterogeneity and sensitivity of spatial epidemic patterns to changes in several underlying parameters (behavioral and disease related)^22^. In general, the resultant patterns are fairly intricate, demanding more refined mechanisms going beyond simple gravity-based interactions or a wave-like diffusion^2,20,23–25^. when in real-world epidemics human mobility may not agree with these standard predictions^26^. In many cases these more refined models have considered time variation of the underlying network^27–29^. In related work, a mathematical model of the spatial development of social disorder, treated as a contagion, was shown to simulate emergent spatial patterns, while considering the distances travelled to riot locations and the deterrent effect of policing^30^. Importantly, this spatial interaction model utilized the concept of the rational offender contemplating the potential utilities (rewards) available at different spatial locations, while minimizing costs and trying to avoid capture. Such examples^30^ highlight that spatial contagion models that address the question of individual mobility would have utility beyond the narrower scope of canonical epidemiological models. Indeed, we may recognise that any evolving social phenomena that shares the underlying contact/interaction basis for spread of a quantity of interest stands to gain from such refinements, with examples of such social dynamics including opinion dynamics, population segregation, social unrest and economic competition, where people may “go mad in herds, while they only recover their senses slowly, one by one”^31^.

The optimal way to model the behaviour associated with individual mobility in such models, however, remains an open question. In this study we aim to provide a rigorous framework that incorporates such dynamic human decision-making into commonly used epidemic/social phenomena models which is capable of explaining and categorizing spatial heterogeneity arising from variable mobility under stress. In doing so usual parameters impacting the evolution of the phenomena (such as the basic reproductive ratio *R*_0_^4,32,33^ in epidemic models), are augmented by parameters accounting for varying levels of rationality.

When such behavior is introduced, cases of particular interest arise when the population contains groups with differing preferences and/or misaligned interests. In such cases the resulting behaviour may lead to several qualitatively distinct solutions, due to the possibility of emergent frustrated dynamics. This may be compared with pattern formations studies carried out in the context of vegetation models in ecology^34,35^, prey-taxis and predator avoidance systems^36–41^ and other similar diffusive processes. This may lead to the existence of phase transitions between distinct regimes of behavior in a space of control parameters. Population groups with varied compartment dependent^42,43^ and mobility dependent behaviors have appeared in related models^30,44^, but to our knowledge critical phenomena have not been systematically investigated in such contexts. In identifying such transitions we adopt approaches from statistical physics, such as percolation theory and information-theoretic measures of criticality.

The study of critical dynamics more broadly is of great importance to epidemiology, complex systems and network processes. For example, slowly varying an underlying control parameter, such as the transmissibility of the disease, may induce a sudden change in an observable order parameter, such as the disease attack rate^4,45^. Indeed, it has been argued that a maladapted pathogen, initially with ${R}_{0} < 1$, can lead to an epidemic if genetic variations, events such as crossing the species barrier, or changes in the host population cause *R*_0_ to exceed 1^46^. Other studies indicate that tracing an approach towards an epidemic threshold can also improve prediction and prevention of epidemics^47^. Of particular relevance is the study of Balcan and Vespignani^48^, wherein the consideration of human mobility and activity patterns on the spread of infectious diseases lead to “a phase transition between a regime in which the contagion affects a large fraction of the system and one in which only a small fraction is affected”.

The modelling framework we present is based on the Boltzmann-Lotka-Volterra (BLV) methodology^49^. The dynamics underpinning the framework are comprised of a fast- and slow-scale component reflecting (i) the preference for temporary relocation and minimizing travel cost, and (ii) the underlying dynamics of transmission and recovery (here implemented as a Susceptible–Infectious–Susceptible (SIS) model), respectively. The fast-scale dynamics are modeled using the Maximum Entropy (MaxEnt) method which yields the mobility flow with the least bias given incomplete information about the system. Both the travel impedance and the preference for temporary relocation to safer locations are quantified through Lagrange multipliers interpreted as coefficients capturing the bounded rationality of the affected individuals. The mobility flow is then re-evaluated dynamically, affecting a non-linear feedback mechanism in the slower-scale SIS model, defined across a network. Importantly, we identify several distinct phases of spatial patterning in the endemic state of the epidemic, dependent on the rationality parameters of the compartment populations, identifying critical regimes and characterizing the system through phase diagrams.

The proposed approach to modelling population mobility in the presence of a severe disease or social disorder, is highly flexible, straight-forwardly adapting to various compartmental models, network topologies and utility functions. It thus serves as an ideal test bed for investigating, within a single setting, various factors affecting individuals’ choice, so that the critical phenomena emerging in response to changes in human behavior can be understood. This addresses questions such as (i) when do small changes in the population’s perception of the epidemic risks, or social disorder threats, trigger abrupt and significant changes in the spatial distribution of people across the affected areas, (ii) what is the extent of mixing within the affected communities, and (iii) under what conditions are there co-existing stable and unstable regions within a larger affected territory.

## Methods

Here we present our framework designed to extend common epidemiological/social models. Specifically, we generalise the SIS-network model^50,51^, described in SI Appendix Section 1, to incorporate dynamic mobility that responds to the evolving state of the epidemic and which allows for compartment-dependent behavior. This is in contrast to existing models which assume temporal and compartmentally homogeneous population mobility^50,51^. Although more complex compartmental models such as Susceptible-Infected-Recovered-Susceptible (SIRS) models could be considered under this framework with state dependent mobility occurring only if an individual has been previously infected, the steady state behaviours of these models will be similar, with a well defined endemic equilibrium which is locally asymptotically stable^52,53^, with the primary difference being the transient behaviour of the model. As our analysis focuses on the potential of this framework and the long term behaviour of the model, there is little additional benefit to considering more complex compartmental models for the analysis presented in this work. However, in the case of analysis to specific data, these extensions would be necessary. The structure of the model is informed by the Boltzman-Lotka-Volterra (BLV) framework^49,54^, consisting of a slow dynamic, the infection dynamics captured by the SIS-network model^50,51^, and a fast dynamic, the dynamic, state dependent mobility terms.

The SIS-network model considers $M$ locations, each with its own sub-population of ${N}_{i}$, $i\in \{1,\ldots ,M\}$, individuals of which ${I}_{i}$ are infectious and ${S}_{i}={N}_{i}-{I}_{i}$ are susceptible. The state of the epidemic is then characterized by the set of all ${I}_{i}$, written **I**. These locations are organized into a network model which is characterized by a matrix **C** of edge weights ${c}_{ij}$ associated with mobility from location $i$ to location $j$. We note that, in the general case, we may have non-zero diagonal elements ${c}_{ii}$ to specify a cost associated with ‘mobility’ to an individual’s home location. These ${c}_{ij}$ may represent physical distance, effective travel cost etc. according to modelling needs.

We then introduce mobility functions for each compartment of the model: infectious and susceptible. These are denoted ${\phi }_{ij}^{I}({\bf{I}},{\bf{C}})$ and ${\phi }_{ij}^{S}({\bf{I}},{\bf{C}})$, explicitly allowing for network, compartment and infection level dependence in the mobilities. Explicitly, ${\phi }_{ij}^{x}({\bf{I}},{\bf{C}})$ is the fraction of the population of compartment $x$ originating from location $i$ that will mix in location $j$, dependent on the topology **C** and the state of the epidemic **I**. Since these functions change instantaneously in response to any changes in **I** they constitute a fast dynamic assumed to equilibrate on a time scale faster than the evolution of the epidemic. These are brought into the standard SIS-network model such that the progression of the epidemic is given by

1 $$\frac{d{I}_{i}}{dt}=-\,\gamma {I}_{i}+\beta \,\sum _{j,k}\,{\phi }_{ij}^{S}({\bf{I}},{\bf{C}}){\phi }_{kj}^{I}({\bf{I}},{\bf{C}})\frac{{S}_{i}{I}_{k}}{{\hat{N}}_{j}({\bf{I}},{\bf{C}})},$$

where

2 $${\hat{N}}_{j}({\bf{I}},{\bf{C}})=\sum _{k}\,{S}_{k}{\phi }_{kj}^{S}({\bf{I}},{\bf{C}})+{I}_{k}{\phi }_{kj}^{I}({\bf{I}},{\bf{C}}),$$

where ${\hat{N}}_{j}({\bf{I}},{\bf{C}})$ is the total number of individuals mixing at location $j$ (see Eq. 2), $\gamma$ is the individual recovery rate and $\beta$ is the transmission rate between a single infected and susceptible individual within the same location. Consequently, the terms $\beta {\phi }_{ij}^{S}{\phi }_{kj}^{I}{S}_{i}{I}_{k}/{\hat{N}}_{j}$ reflect the rate of total new infections for the susceptible population of $i$ caused by the infected population from $k$ which occur in location $j$ at time $t$. We emphasize that the mobilities ${\phi }_{ij}^{x}({\bf{I}},{\bf{C}})$, $x\in \{S,I\}$, now *vary in time* as the values of the ${S}_{i}$ and ${I}_{i}$ evolve, introducing a qualitatively distinct mechanism for *dynamic* population mobility.

The task which remains is to specify the functional form of these ${\phi }_{ij}^{I}({\bf{I}},{\bf{C}})$ and ${\phi }_{ij}^{S}({\bf{I}},{\bf{C}})$ which could arguably take many different forms. The principle and insight behind the BLV methodology^49,55^ is to recognize that not all choices of these functions are equally reasonable given the limited knowledge of the system available to the modeler. Rather, we should be concerned with identifying the minimal *constraints* that characterize plausible behavior and choose the most likely, or *least-biased*, functional form that is consistent with them. This is the reasoning behind the well-known MaxEnt methodology^56,57^. Consequently, we postulate two fundamental behaviors: (i) a perceived ‘cost’ associated with the graph structure captured by a function of the ${c}_{ij}$ and (ii) a perceived ‘benefit’ associated with the spatial configuration and extent of the epidemic captured by a function of ${I}_{i}$ and ${S}_{i}$. Specifically, here, we consider the cost of mobility from $i$ to $j$ to be given simply by ${c}_{ij}$, whilst the benefit of mobility from $i$ to $j$ to be given by ${b}_{j}={N}_{j}^{-1}({N}_{j}-{I}_{j})$. We note that the cost and benefit functions are not restricted to the functional forms used here and may, in principle, be any reasoned function of the compartment, **C** and **I** or even augmented with additional model details. The MaxEnt method then determines the ${\phi }_{ij}^{x}({\bf{I}},{\bf{C}})$, at each point in time, by maximizing the Shannon entropy of the probability distribution of all individual behavior, such that it is consistent with the current state of the epidemic, **I**, in addition to constraints on the mean benefit due to mobility for infectious and susceptible individuals

3 $${B}^{I}=\sum _{i,j}\,{I}_{i}{\phi }_{ij}^{I}({\bf{I}},{\bf{C}}){b}_{j}/\sum _{i}\,{I}_{i},$$

4 $${B}^{S}=\sum _{i,j}\,{S}_{i}{\phi }_{ij}^{S}({\bf{I}},{\bf{C}}){b}_{j}/\sum _{i}\,{S}_{i},$$

and mean cost of mobility for all individuals

5 $$C=\sum _{i,j}\,({I}_{i}{\phi }_{ij}^{I}({\bf{I}},{\bf{C}})+{S}_{i}{\phi }_{ij}^{S}({\bf{I}},{\bf{C}})){c}_{ij}/\sum _{i}\,({I}_{i}+{S}_{i}\mathrm{)}.$$

Such a solution, detailed in SI Appendix Section 2, is given by

6 $${\phi }_{ij}^{x}({\bf{I}},{\bf{C}}|{\alpha }^{x},\omega )={Z}_{x,i}^{-1}\,\exp \,({\alpha }^{x}{b}_{j}-\omega {c}_{ij}),$$

where ${Z}_{x,i}={\sum }_{j}\,\exp \,({\alpha }^{x}{b}_{j}-\omega {c}_{ij})$. The functional form of ${\phi }_{ij}^{x}$ is entirely determined by the *Lagrange multipliers* ${\alpha }^{x}$ and $\omega$, which correspond to the mean quantities ${B}^{x}$ and $C$. Methodologically, such a formulation provides two distinct modelling perspectives which we illustrate in (7). In the first, ${B}^{x}$ and $C$ could be measured from data, from which the ${\alpha }^{x}$ and $\omega$ would be inferred allowing the least biased fit to data^58^. In this case the ${\phi }_{ij}^{x}$ would constitute the minimal *empirical* model consistent with the observables. In contrast, the second, which is considered in this work, considers the relationship in the other direction. In this case ${\alpha }^{x}$ and $\omega$ are considered to be free model parameters corresponding to different strengths of behavior in response to concepts of cost and benefit.

7 $$(\begin{array}{c}{\alpha }^{S}\\ {\alpha }^{I}\\ \omega \end{array})\mathop{\mathop{\leftrightharpoons }\limits^{{\rm{S}}{\rm{p}}{\rm{e}}{\rm{c}}{\rm{i}}{\rm{f}}{\rm{y}}\,{\rm{c}}{\rm{o}}{\rm{n}}{\rm{s}}{\rm{t}}{\rm{r}}{\rm{a}}{\rm{i}}{\rm{n}}{\rm{t}}{\rm{s}}:\,{\rm{I}}{\rm{n}}{\rm{f}}{\rm{e}}{\rm{r}}\,{\rm{m}}{\rm{o}}{\rm{d}}{\rm{e}}{\rm{l}}\,{\rm{p}}{\rm{a}}{\rm{r}}{\rm{a}}{\rm{m}}{\rm{e}}{\rm{t}}{\rm{e}}{\rm{r}}{\rm{s}}}}\limits_{{\rm{S}}{\rm{p}}{\rm{e}}{\rm{c}}{\rm{i}}{\rm{f}}{\rm{y}}\,{\rm{p}}{\rm{a}}{\rm{r}}{\rm{a}}{\rm{m}}{\rm{e}}{\rm{t}}{\rm{e}}{\rm{r}}{\rm{s}}:\,{\rm{M}}{\rm{o}}{\rm{d}}{\rm{e}}{\rm{l}}\,{\rm{g}}{\rm{e}}{\rm{n}}{\rm{e}}{\rm{r}}{\rm{a}}{\rm{t}}{\rm{e}}{\rm{s}}\,{\rm{q}}{\rm{u}}{\rm{a}}{\rm{n}}{\rm{t}}{\rm{i}}{\rm{t}}{\rm{i}}{\rm{e}}{\rm{s}}}(\begin{array}{c}{B}^{S}\\ {B}^{I}\\ C\end{array})$$

Different choices thus correspond to distinct, hypothetical, *model* populations with different behaviours which we can analyze. In thermodynamic contexts, in which MaxEnt techniques were first conceived^56,57^, the Lagrange multipliers appear as intensive parameters such as temperature. Analogously, the two modelling perspectives would amount to i) inferring the temperature given data on measured energies and ii) generative modelling of hypothetical situations at different temperatures. In practical terms, each of these perspectives amounts to a distinct way to use this modelling framework. Firstly, a theoretical model based on assumptions around the effects of disease presence on mobility could be compared to real data in order to test hypothesis around state dependent mobility or identify likely outcomes of an epidemic. Conversely, if time series for mobility and infection dynamics exist on the same resolution, the mean quantities could be calculated and used in order to infer the behavioural parameters of different countries, indicating the extent to which individuals mobility is impacted by the presence of disease. The combination of these approaches would allow for the inference of parameters to be performed on historical data (second approach) and the parameters generated in this way could then be used for future forecasting of disease within similar communities (first approach).

**Figure 1:**
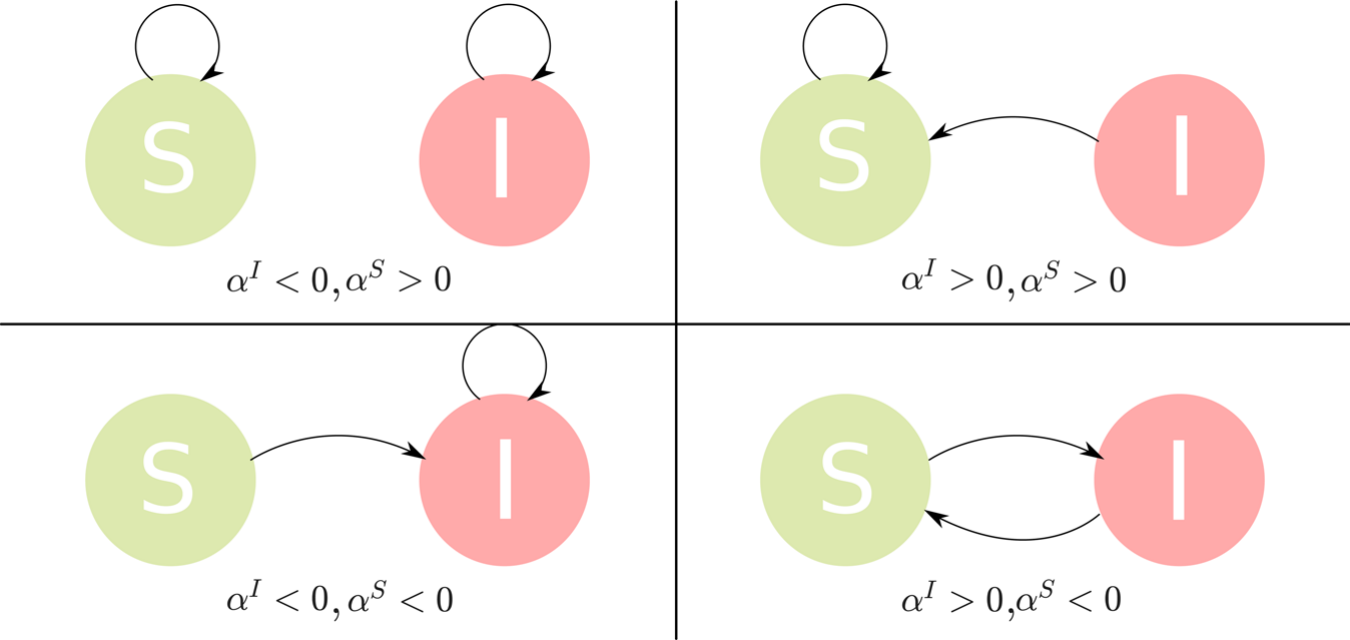
Dominant mixing preferences within each quadrant of the ${\alpha }^{I},{\alpha }^{S}$ phase space. The larger the magnitude of ${\alpha }^{I}$ or ${\alpha }^{S}$ the more dominant the mixing behaviour. The mixing of each quadrant from top right to bottom right counter-clockwise may be used to describe: typical epidemic behaviour where all individuals avoid infectious individuals (top right), Schelling style segregation where individuals actively seek similar individuals (top left), spreading of social myths where individuals who are aware of the myth continue to associate with those who do (bottom left) and rioting behaviours (bottom right).

This formulation is entirely consistent with behavioral models that consider individuals with bounded rationality^59,60^. In such a formulation an objective pay-off function is proposed which is then paired with a rationality parameter. Here the objective pay-off functions are the benefit ${b}_{j}$ and cost ${c}_{ij}$ with corresponding rationality parameters ${\alpha }^{x}$ and $\omega$. Given a fixed $\omega$ we may consider a two dimensional rationality phase space $\{{\alpha }^{S},{\alpha }^{I}\}\in {{\mathbb{R}}}^{2}$ such that ${\alpha }^{S}$ and ${\alpha }^{I}$ may be positive or negative capturing a range of possible behaviours. The degree of rationality is captured by $|{\alpha }^{x}|$ with the benefit ${b}_{j}$ being perceived as negative/positive compartment $x$ based on the sign of ${\alpha }^{x}$. I.e. when ${\alpha }^{x} > 0$, compartment $x$ views low infectiousness as a benefit and so higher rationality increases mobility towards locations with low levels of infection. On the other hand when ${\alpha }^{x} < 0$, compartment $x$ views low susceptibility as a benefit and higher rationality increases mobility towards locations with relative low levels of susceptible individuals, see Fig. 1. When the $|{\alpha }^{x}|$ is infinite the pay-off function entirely determines behavior associated with that pay-off, with all individuals at location $i$ choosing the option(s) which maximize/minimize the payoff ${\sum }_{j}\,{\phi }_{ij}^{x}({\bf{I}},{\bf{C}})\,{b}_{j}$. In contrast, when $|{\alpha }^{x}|=0$, behavior is indifferent to the pay-off such that there is a flat distribution over all options. For the purposes of this study we insist that $\omega  > 0$ such that edge weights on the network are always considered as a (positive) cost. We emphasize that the above formulation insists on homogeneous statistical behavior in the individuals, since the constraints are in terms of averages over the total population, however this assumption can be relaxed as discussed in SI Appendix Section 2.

Finally, we discuss the model parameters utilized in this study. We emphasize that our methodology, as presented, can operate on any graph structure, with topologically inhomogeneous behavior, and incorporated into any existing compartmental metapopulation model on a graph. However, in order to present, in a transparent and straight-forward way, the possible richness in behavior such a methodology can produce we restrict ourselves to the SIS model with topologically homogeneous behavior on a simple square lattice of length $L$, such that we have $M={L}^{2}$ locations, with periodic boundary conditions. At each location we consider an arbitrary, normalized, population ${N}_{i}=1$. Elements ${c}_{ij}$ of **C** are taken to be $1$ for the self interaction term ${c}_{ii}$ in addition to those connecting each location to its four immediate neighbors and $\infty$ otherwise such that mobility from site $i$ only occurs to itself and its topological neighbors. Since all finite ${c}_{ij}$ are equal the choice of $\omega$ is arbitrary so long as $0 < \omega  < \infty$, simplifying the analysis in this case.

## Results

Here we investigate the behavior of the model specified in the previous section. Whereas typical epidemic modelling investigates the qualitative extent and reach of epidemics given a phase space of infection and recovery rates (*β* and *γ*), we consider a phase space of differing compartmental behavior through varying values of the rationality parameters ${\alpha }^{S}$ and ${\alpha }^{I}$, with a focus on the regimes of emergent spatial structure in the endemic infection levels.

To do so we focus primarily on the post-critical regime (${R}_{0}=\beta /\gamma  > 1$), comprehensively exploring the rationality phase space defined by ${\alpha }^{S}$ and ${\alpha }^{I}$ with fixed infection and recovery rates $\beta =10$ and $\gamma =5$, allowing us to consider a wide range of potential behaviours. Very broadly we can divide these behaviours into four main types which we associate with the four quadrants of the rationality phase space, demonstrated in Fig. 1 where each quadrant is defined by the signs of the $\alpha$ parameters. The positive quadrant (${\alpha }^{S},{\alpha }^{I} > 0$) represents plausible mixing preferences present in epidemics where all individuals predominantly avoid infectious individuals. The second quadrant (${\alpha }^{I} < 0,{\alpha }^{S} > 0$) possesses dominant mobility behaviour similar to Schelling style segregation^61^ where individuals prefer to mix with members of their own group ($S$ or $I$). In the third quadrant (${\alpha }^{I} < 0,{\alpha }^{S} < 0$) all individuals consider ‘infection’ as a benefit and seek out the infected population. This type of mixing behavior may be of particular use in the modelling of the spread of certain social phenomena, e.g. rumors, social myths etc. Finally, in the fourth quadrant (${\alpha }^{I} > 0,{\alpha }^{S} < 0$), individuals seek members of the opposite group. This behaviour is much more dynamic and unstable, suggesting application to more extreme social contexts such as rioting and social unrest.

**Figure 2:**
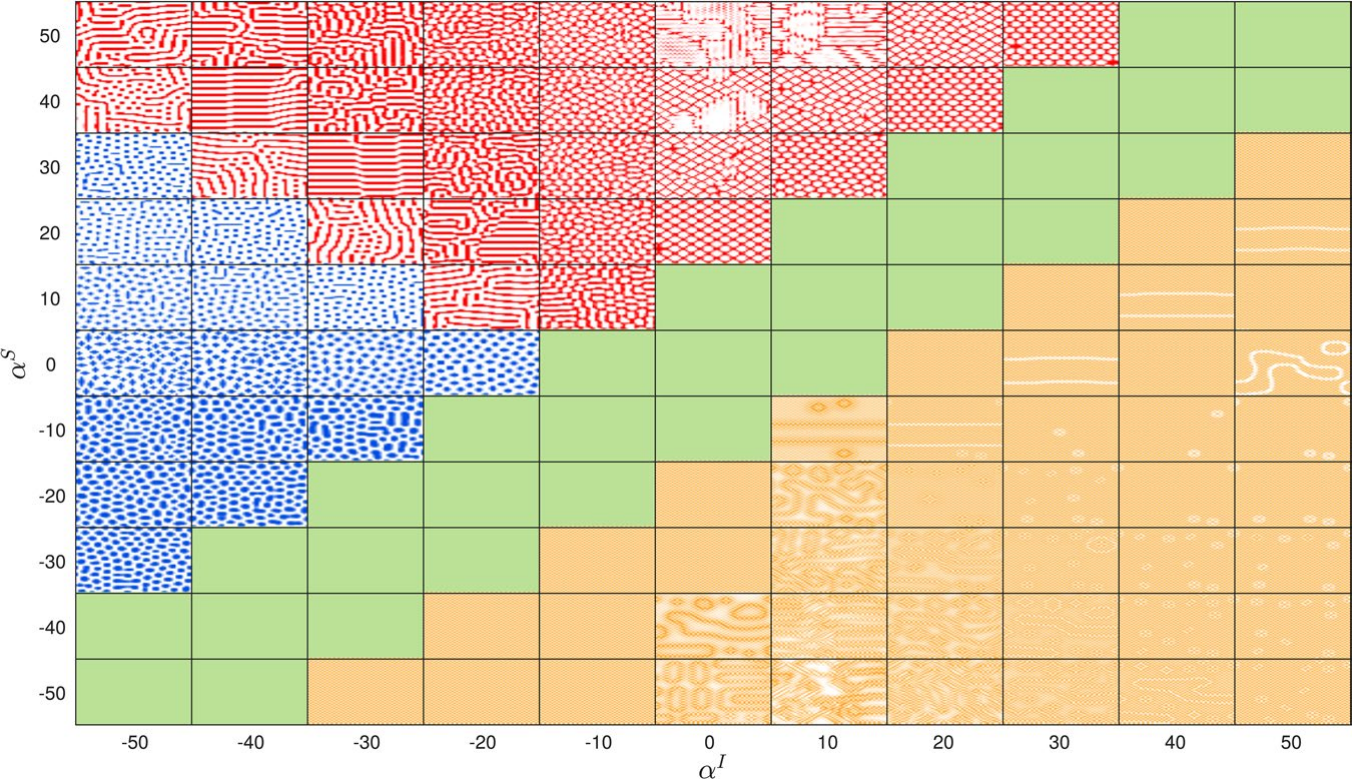
Simulation snapshots after 20000 timesteps with Δ$t=0.001$ for $\beta =10$, $\gamma =5$ $\omega =1$ on a $50\times 50$ lattice demonstrating qualitatively different phases across a phase space defined by ${\alpha }^{S}$ and ${\alpha }^{I}$. Region (i), the unseparated regime, is coloured green, region (ii), the connected regime, is coloured red, region (iii), the isolated regime is coloured blue whilst region (iv) the anti-aligned regime is coloured in orange. Each snapshot is renormalised by the infection mean and standard deviation to emphasise key spatial differences. Darker areas represent regions of higher relative infection.

We integrate Eq. 1 using a forward Euler method with Δ$t=0.001$. Since the equations are deterministic the ensemble of behavior given specified model parameters derives entirely from the ensemble of initial conditions which we specify to be an i.i.d uniform random initial infectious population, ${I}_{i}$, for each location on $[0,0.05]$. Evolution of the dynamics leads to stable long term solutions with distinct typical spatial structures dependent on the rationality parameters, despite the uniform topology. Figure 2 illustrates these behaviors with data from direct simulation. We observe four qualitatively distinct regions: (i) a flat spatial distribution of infection (coloured green) which we refer to as the ‘unseparated regime’, (ii) large highly connected domains (coloured red) which we refer to as the ‘connected regime’ (iii) many small disconnected domains (coloured blue) which we refer to as the ‘isolated regime’ and (iv) an alternating checkerboard pattern containing long-lived defect structures due to initial heterogeneity (coloured orange), which we refer to as the ‘anti-aligned’ regime. We mention that the regimes observed in the space defined by ${\alpha }^{S} > 0$ and ${\alpha }^{I} < 0$ are reminiscent of the standard sequence of spatial patterns (gaps, labyrinth, spots) observed in vegetation growth models^34,35^.

It is important to note, however, that these regimes do not map directly onto the four quadrants laid out in Fig. 1. For example, in the first quadrant, expected to be most relevant for conventional epidemic modelling, we find qualitatively different spatial patterns in the endemic state despite the entire population (both $S$ and $I$) sharing a consistent conception of benefit (i.e. ${\alpha }^{S} > 0$ and ${\alpha }^{I} > 0$), through quantitative differences in their rationality $(|{\alpha }^{I}|$ vs. $|{\alpha }^{I}|)$.

The existence of these distinct regimes is highly suggestive of a system with several stable phases separated by *phase transitions* where the system undergoes large re-configurations in response to very small variations in system parameters. In order to characterize these regimes, we seek to understand the qualitative picture presented in Fig. 2 mathematically. In doing so we offer evidence that such regimes are indeed well characterized as distinct configurational phases separated by discontinuous transitions. We present three order parameters designed to distinguish between the salient features in each of the four identified regimes. We then investigate the effect that changes in the basic reproductive ratio have upon the phase diagram.

**Figure 3:**
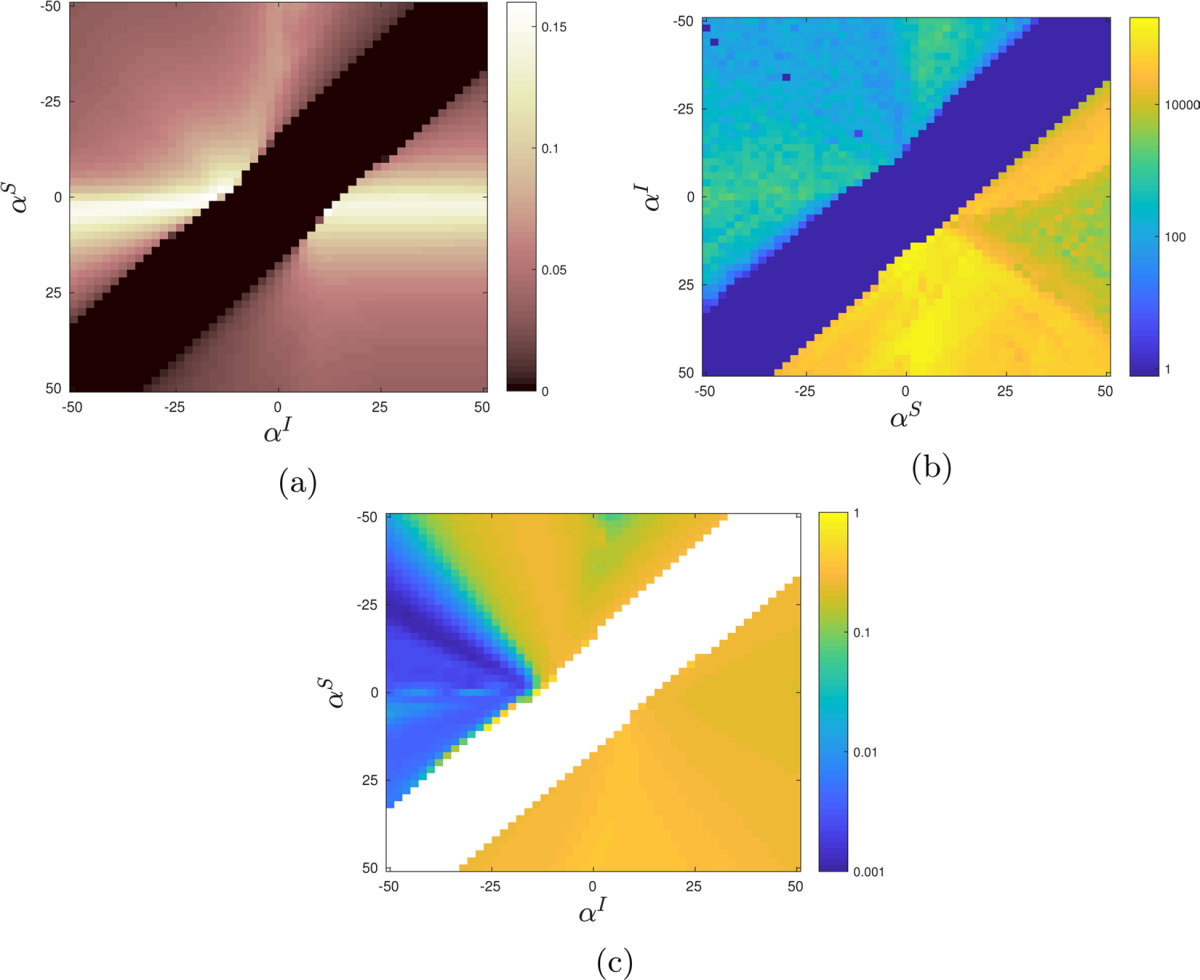
Heatmaps of each of the order parameters (**a**) standard deviation *σ*, (**b**) staggered magnetisation ${M}_{N}$ and (**c**) percolation order parameter $\langle \hat{r}\rangle$ for $\beta =10$, $\gamma =5$, $\omega =1$ on a $50\times 50$ lattice averaged across 50 runs. A vanishing standard deviation (subfigure (**a**)) is associated with the unseparated regime whilst positive standard deviations are associated with phase separation (the connected, isolated and anti-aligned regimes).We note the largest standard deviations occur for $|{\alpha }^{S}|\approx 0$ when the movement of the susceptible population occurs irrespective of the presence of infection. The staggered magnetisation (subfigure (**b**)) distinguishes the anti-aligned regime (yellow) from the other phase separated regimes (light blue) and the unseparated regimes (dark blue). $\langle \hat{r}\rangle$ (subfigure (**c**)) can be used to distinguish two distinct phases in the upper left quadrant previously referred to as the connected regime (yellow region) and the isolated regime (blue region). $\langle \hat{r}\rangle$ is undefined in the white regions (the unseparated regime).

First, we consider the distinction between the unseparated regime and the connected/isolated/anti-aligned regimes. The behavior in the unseparated regime is qualitatively identical to SIS-network models without state dependent mobility, with the infection levels being topologically homogeneous. However, the connected, isolated and anti-aligned regimes exhibit behavior not observed in conventional network models, with regions of relative high and low infection levels forming stable domain-like structures. As either rationality parameter increases we observe a rapid reconfiguration from a singular distribution of infection levels over all locations to one with substantial variance with two identifiable peaks. Such a distinction can be compactly characterized by the standard deviation of the distribution, $p({I}_{i})$, functioning here as an effective order parameter^62^. Figure 3a illustrates the discontinuous change in standard deviation from zero to positive values at the region in phase space separating the unseparated and connected/isolated and anti-aligned phases. This discontinuous behavior is extremely sensitive to changes in ${\alpha }^{I}$ and ${\alpha }^{S}$, even at low system sizes. This is indicative of an order/disorder phase transition where the unseparated regime corresponds to the homogeneous ordered phase and the connected/isolated/anti-aligned regimes correspond to the disordered phase.

Secondly, we consider the distinction between the phase separating behaviour found in the ${\alpha }^{S} < {\alpha }^{I}$ and ${\alpha }^{S} > {\alpha }^{I}$ regimes. We proceed by utilising the staggered magnetisation, commonly used to study anti-ferromagnetic systems, to identify the underlying checkerboard structure of the anti-alignment observed in the ${\alpha }^{I} > {\alpha }^{S}$ region. The staggered magnetisation compares a system to an ideal anti-ferromagnetic system, a Néel anti-ferromagnet^63^, which in our case, consists of an $L\times L$ two dimensional grid of alternating +1 and −1 values. Given a standard raster ordering of the locations, such that the *m*-th and *n*-th co-ordinates in the $x$ and $y$ dimensions map to the $(m+nL)$-th location, the staggered magnetisation ${M}_{N}$ for our epidemic system is thus defined as

8 $${M}_{N}=|\mathop{\sum }\limits_{x=0}^{L-1}\,\mathop{\sum }\limits_{y=0}^{L-1}\,{(-1)}^{x+y}{I}_{i}|,\,i=x+yL.$$

Figure 3b demonstrates that the staggered magnetisation ${M}_{N}$ is capable of distinguishing the anti-aligned phase from the other phase separated regimes. This is due to the strong checkerboard pattern of higher and lower infection levels which most closely resembles the Néel anti-ferromagnet. We observe this to hold despite the presence of boundary defects between domains of equally stable, but incompatible configurations of the checkerboard pattern. In contrast, in the other phase separated regimes where we observe larger domains forming ‘dotted’ or ‘snaking’ patterns, locations are much more likely to have a similar level of infection to their neighbors, resulting in a significantly lower staggered magnetisation.

**Figure 4:**
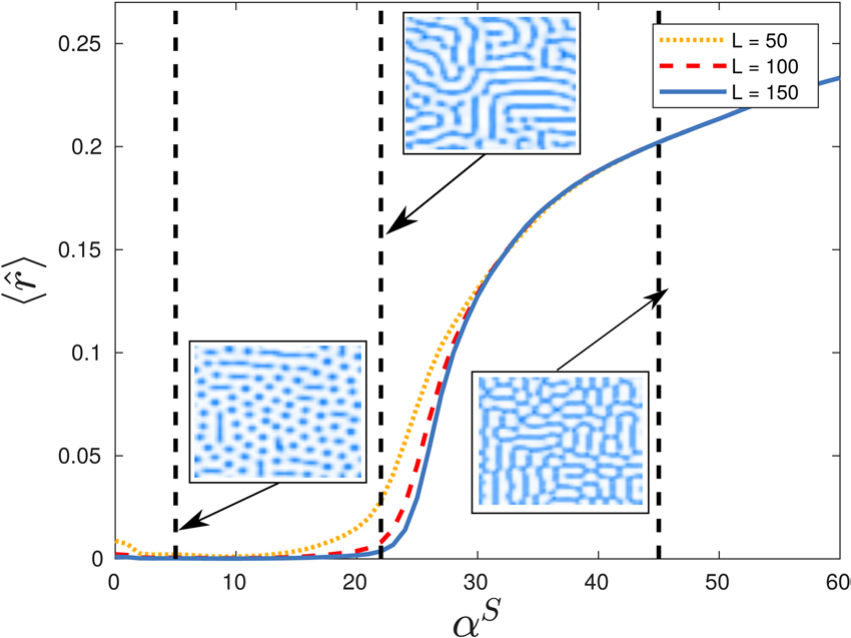
Normalized mean cluster size of a randomly selected location $\langle \hat{r}\rangle$ as a function of ${\alpha }^{S}$ for ${\alpha }^{I}=27.5$ with $\beta =10$, $\gamma =5$, $\omega =1$, on three different lattice sizes. This slice across ${\alpha }^{I}=-\,27.5$ intersects the connected and isolated regimes. Inset are simulation snapshots on a $50\times 50$ lattice after 20,000 timesteps with Δ$t=0.001$ for three values of ${\alpha }^{S}$ which demonstrate examples of the spatial patterns observed in regions (**a**) ${\alpha }^{S} < 20$ (the isolated regime), (**b**) $20 < {\alpha }^{S} < 30$ (near criticality), (**c**) ${\alpha }^{S} > 30$ (the connected regime).

The distinction between the connected and isolated regimes is more nuanced since the transition occurs between two phase separated populations with broad distributions of infection levels. Consequently, we instead turn to measures of percolation^64–66^ to characterize the regimes using statistics of cluster formations within the emergent structures. For the purposes of this study we define a cluster as a contiguous region of sites with infection levels above the population mean value. In order to avoid artifacts related to the square topology we consider sites to be contiguous to one another if they are laterally or diagonally adjacent such that each location is contiguous to eight others. Once clusters are identified the system is amenable to the methods used to study percolation on binary lattices^67^. A key quantity in such studies is the probability that a selected site belongs to a cluster of size *r*, $p(r)$, which can be normalized by considering $\hat{r}=r/{L}^{2}$. A related order parameter from the study of percolation on such lattices, $\langle \hat{r}\rangle$, is illustrated, where relevant, over the rationality phase space in Fig. 3c. Such a measure is undefined for the unseparated regime due to its singular distribution, but the connected and isolated phases show very noticeably different values of this percolation order parameter with a separating boundary running diagonally through the phase space. This transition is much smoother than that bounding the unseparated phase. Consequently, in Fig. 4, we demonstrate increasingly rapid growth of $\langle \hat{r}\rangle$, and thus emergence of the connected phase, as ${\alpha }^{S}$ is increased along a line of constant ${\alpha }^{I}=-\,27.5$ through the rationality phase space, as larger lattice sizes are considered and finite size effects become negligible. Inset are typical configurations associated with the order parameter $\langle \hat{r}\rangle$ illustrating the rapid onset of system wide connected regions of high infection at higher levels of ${\alpha }^{S}$ from local, isolated, regions at lower ${\alpha }^{S}$.

**Figure 5:**
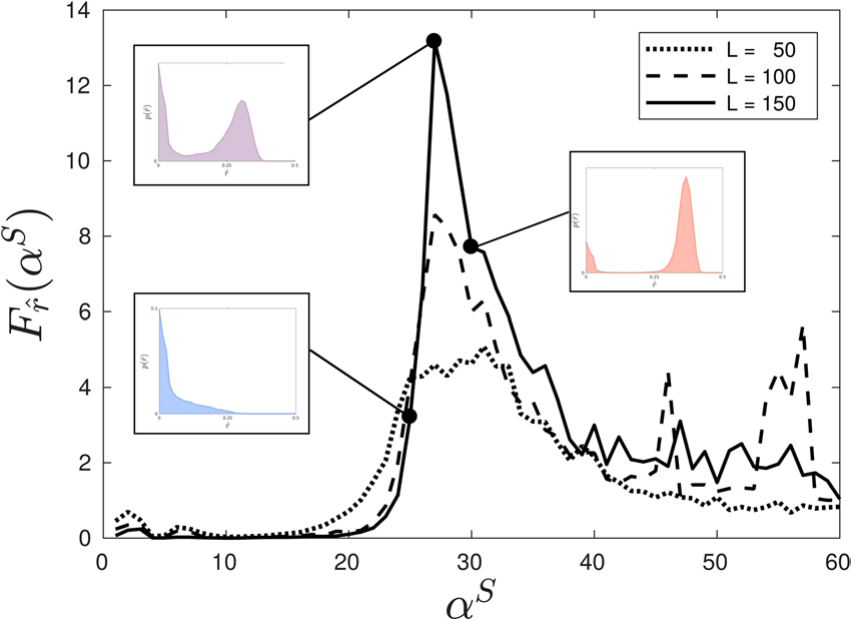
Fisher information of $\hat{r}$ with respect to ${\alpha }^{S}$, the probability that a randomly selected site belongs to a cluster of normalized area $\hat{r}$. We compute ${F}_{\hat{r}}({\alpha }^{S})$ for ${\alpha }^{I}=-\,27.5$, $\beta =10$, $\gamma =5$, $\omega =1$ on three different lattice widths $L$. The distribution for calculating the Fisher Information is an aggregate of 5000 time series from randomized initial conditions with an identical number of bins. We observe that the maximal Fisher information corresponds with the maximal growth in $\langle \hat{r}\rangle$ shown in Fig. 4.

The existence of a phase transition between these two regimes can also be observed in model free measures of criticality, namely the Fisher information which is proven to diverge at a phase transition^68–71^. This divergence is due to its direct relationship to the rate of change of a corresponding order parameter^68^. The Fisher information is defined as the expectation

9 $${F}_{X}(\theta )=E[{({\partial }_{\theta }\log P(X;\theta ))}^{2}],$$

where for the present system $X={\bf{I}}$. However, the full phase probability distribution $P({\bf{I}};{\alpha }^{x})$ is impractical to work with, due to its high dimensionality, so instead we exploit the inequality ${F}_{T}(\theta )\le {F}_{X}(\theta )$, where $T$ is a statistic of $X$ with equality holding if and only if $T$ is a sufficient statistic of $X$. Then, under some relatively weak assumptions, a divergence in ${F}_{T}(\theta )$ implies a divergence in ${F}_{X}(\theta )$ and thus the existence of a phase transition. Utilizing $\hat{r}$ as a statistic of **I**, we compute the Fisher information of $\hat{r}$ about ${\alpha }^{S}$, ${F}_{\hat{r}}({\alpha }^{S})$, for the same line of constant ${\alpha }^{I}=-\,27.5$ as in Fig. 4 with increasing lattice sizes, illustrated in Fig. 5. As the lattice size increases the Fisher information of the statistic $\hat{r}$ peaks at larger and larger values indicative of a divergence in the infinite system limit and thus a phase transition. Shown inset are the distributions $p(\hat{r})$ demonstrating a rapid change in configurations across the transition, with a concentration of probability at small cluster membership at lower ${\alpha }^{S}$ and a concentration of probability at large cluster membership at higher ${\alpha }^{S}$.

**Figure 6:**
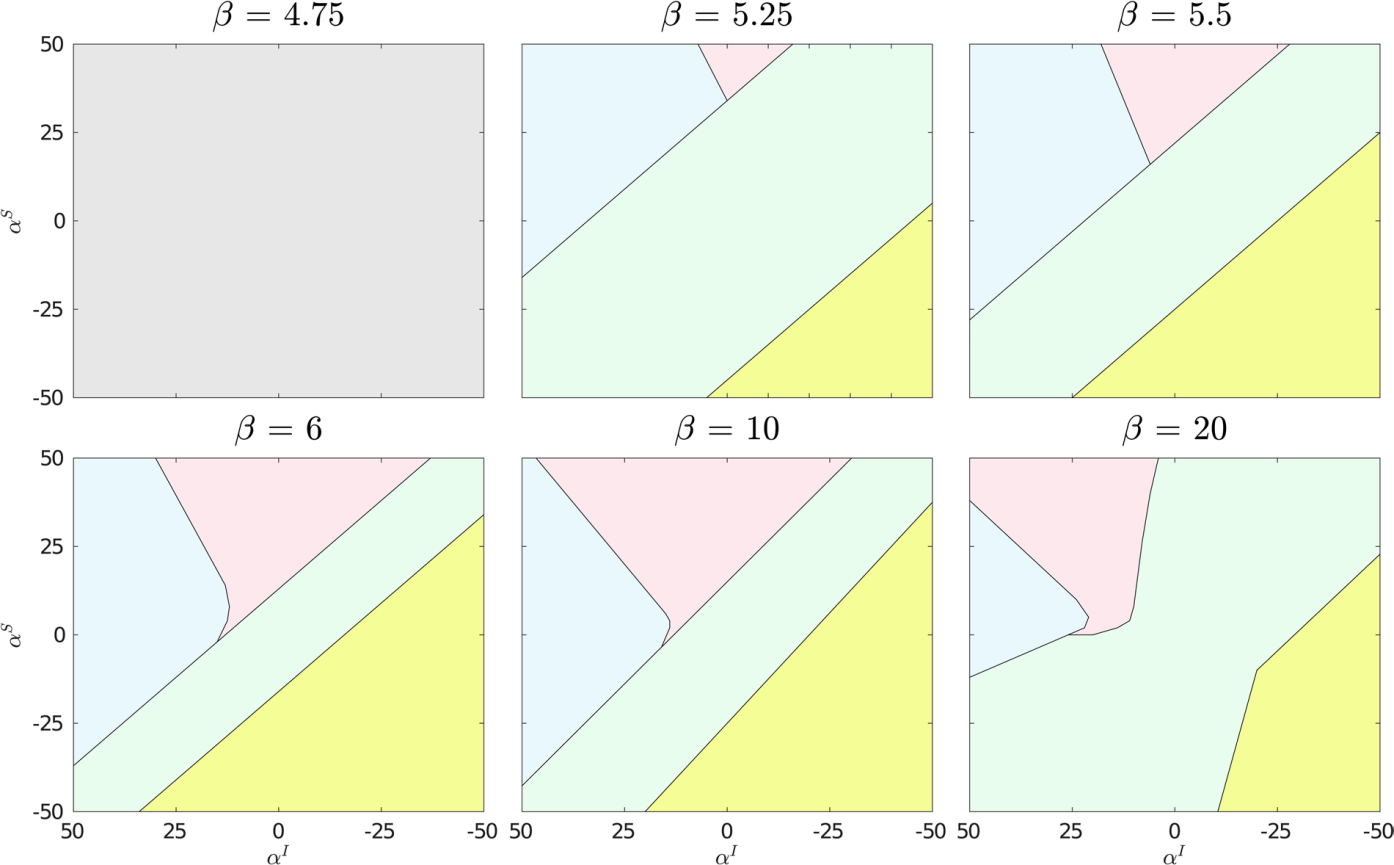
Phase diagrams for multiple values of $\beta$ for $\gamma =5$, $\omega =1$ on a $50\times 50$ lattice. The region in grey corresponds to the unseparated regime below the critical epidemic threshold. Regions in green correspond to unseparated regime, with endemic infection levels. Yellow regions correspond to the anti-aligned phase. Blue regions correspond to the isolated regime and red regions correspond to the connected regime. The majority of the phase separating behaviour in the assortative mixing case when ${\alpha }^{S} > 0$ and ${\alpha }^{I} < 0$.

Finally, we demonstrate the interaction between the canonical parameters influencing the endemic infection levels of an epidemic under the SIS compartmental model, namely $\beta$ and $\gamma$, and the behavioral parameters ${\alpha }^{S}$ and ${\alpha }^{I}$. This is achieved by varying $\beta$ from pre-critical to post-critical values given fixed $\gamma =5$ and determining the resulting phase diagrams in the behavioral space using order parameters $\sigma$, $\langle \hat{r}\rangle$ and ${M}_{N}$. The results are shown in Fig. 6. For $\beta  < \gamma$ we observe no spatially inhomogeneous behavior, consistent with the absence of an epidemic at pre-critical parameter choices. It is known that such systems will have a typical infection phase transition independent of its topology at $\beta /\gamma =1$^50^ and accordingly the system sustains endemic infection levels uniformly at $\beta =5$ for all values of ${\alpha }^{S}$ and ${\alpha }^{I}$.

As $\beta$ is increased further, the phase separated regimes (isolated/connected/anti-aligned) grow in size until for very large values of $\beta$ the phase space is dominated by the unseparated phase due to the high levels of infection which saturate the dynamics. Consequently we observe that at low (but post-critical) and high $\beta$, a large discrepancy between ${\alpha }^{S}$ and ${\alpha }^{I}$ is required to observe phase separation, but at intermediate values separation appears at much smaller differences in population behavior.

## Discussion

In this article we have proposed and investigated a dynamic spatial interaction model applicable to studies of epidemics and social disorders. The model explicitly allows for state dependent mobilities which co-evolve with the underlying phenomenon leading, here, to a rich range of behaviors not observed in models without this feature.

This model offers two significant contributions, one methodological and one phenomenological.

Methodologically, we have incorporated the BLV framework, that has independently found success in modelling social phenomena ranging from urban and economic growth^55,72–75^ to ecological or crime dynamics^30,76^, into the framework of epidemic modelling. The application to epidemic modelling was first postulated by Wilson^49^, which has since remained an unexplored avenue of investigation. This methodology has several important benefits to the study of social phenomena where explicit mechanisms remain elusive yet a need for plausible models exist. It achieves this by constructing distributions of behavior that include the minimum number of assumed extraneous features which also match the data and/or underlying phenomena at hand, often characterized as being the ‘least-biased’. Given a set of constraints from data or well rationalized utility functions, the implication is that any other choice introduces unjustified assumptions into the model. It is also highly flexible due to its close relationship to economic rationality models and the relative simplicity of modifying or augmenting the set of utility functions which determine the values and number of Lagrange multipliers. In turn the framework is trivial to incorporate into any compartment model, not just the SIS dynamics considered here.

Finally, we discuss the phenomenological implications resulting from the results we have presented. We have implemented a model dynamic that allows for modified individual behavior in response to the evolving state of the epidemic or social phenomena. This may be particularly relevant in cases of severe outbreaks, especially those that lead to or coincide with civil unrest where mobility patterns may be maximally influenced by pertinent local factors rather than regular travel patterns. A consequence of such a dynamic is the emergence of distinct phases related to the spatial structure of infectious spread in the endemic steady state. These regimes are separated by sharp transitions in the rationality phase space. As with the sharp onset of disease spread at the epidemic phase transition, such a phenomenon is of great interest and importance as it implies that large scale, qualitatively distinct, behavior can be highly sensitive to very small changes in human decision-making. Significantly, we observe such phenomena even in cases where the dominant mixing preferences are identical but only the relative levels of rationality ($|{\alpha }^{S}|$ and $|{\alpha }^{I}|$) differ. For instance our results imply that a population where all individuals exhibit infection avoiding behaviour may still exhibit phase separation should the rationality parameters of the susceptible and infectious individuals differ sufficiently.We emphasize that in the proximity of these thresholds, small changes in the perception of local risks and benefits experienced by human decision-makers can trigger sudden and significant changes in the global system behavior. These changes can be of importance to real world applications such as the rapid (re-)deployment of medical teams however calibration of these models to real-world data sets is required to produce detailed recommendations.

Further work may focus on the extension of such a model to more complicated compartmental models, such as Susceptible-Infected-Recovered-Susceptivle (SEIRS) or Susceptible-Exposed-Infected-Recovered-Susceptivle (SEIRS) compartmental models, where infection avoidance behaviours could be considered only for individuals who have been infected in the past, allowing for an in depth analysis of the range of transient behaviours present in such a model. Additionally, we may consider more realistic topologies including more complicated spatial structures and/or include long range, migration-like, transport of individuals across the system.

## Supplementary information

Supplementary Information.
