# Supplementary material for: Population mobility induced phase separation in SIS epidemic and social dynamics
Source: Sci Rep. 2020 May 6;10:7646. doi: 10.1038/s41598-020-64183-1 (PMC7203161; doi:10.1038/s41598-020-64183-1)
Supplement: Supplementary file 1 — Supplementary Information. [file 41598_2020_64183_MOESM1_ESM.pdf]

Population mobility induced phase separation in SIS epidemic and social dynamics – SI Appendix

Nathan Harding, Richard E. Spinney and Mikhail Prokopenko

1 SIS-network epidemic model

One of the corner-stones of mathematical epidemiology is compartmental modelling. In these models sections of the population are recognized as being in one of several distinct states dependent on the individuals' infection status. Dynamics are then introduced to account for how the population may move between these states through time. In the simplest case there may be only two compartments, Infectious and Susceptible, relating to individuals who are currently harbouring the disease (infected and capable of transmitting it) and those who are not and are thus at risk of being infected. In the SIS model, upon clearing an infection, infectious individuals return to the susceptible state, and so can be re-infected [1]. Many extensions are possible, by elaborating on mutually exclusive compartments, for example, adding Recovered state with an acquired immunity in the SIR model [2] (typically used for EVD or influenza modelling).

The SIS model has the following form:

$$\frac{dI}{dt} = \frac{\beta IS}{N} - \gamma I \tag{1}$$

and

$$S + I = N \tag{2}$$

where β is a parameter describing the rate of infection between an infectious and susceptible individual, and γ is a parameter describing the rate at which infectious individuals recover from infection. These dynamics produce a typical solution ("epidemic curve") which exhibits rapid growth in the number of infectious individuals from an initially low level. Eventually the system reaches a steady state where the global rates of recovery and infection are equal, i.e., when $S = \frac{\gamma N}{\beta} = \frac{N}{R_0}$ or $I = 0$. These dynamics can be characterized by the extensively studied parameter, the basic reproductive number (ratio) R_0, defined as the number of infections generated on average by one case over the course of its infectious period, in a susceptible population. For SIS or SIR models there is a critical ("epidemic") threshold $R_0 = 1$, above which there is persistence of infection, and below which epidemics do not develop [3, 4]. Compartmental models can be incorporated into a network model to reflect spatial interactions [5, 6]. Instead of a single N, S and I representing the entire population, one can introduce $N_i, S_i, I_i,\ i = \{1, \ldots, M\}$, which denote the population, the number of susceptible individuals or the number of infectious individuals at location i respectively. Additionally, we consider some fixed mobility/flow ϕ_{ij} representing the proportion of the population of i who, having reached location j, interact with its residents, such that $\sum_j \phi_{ij} = 1$. For example, a multi-city SIS epidemic may be considered, similar to the SIR case studied by Arino and Van den Driessche [6]:

$$\frac{dI_i}{dt} = \beta \sum_k \sum_j \phi_{ij} S_i \frac{\phi_{kj} I_k}{\hat{N}_j} - \gamma I_i, \tag{3}$$

where

$$\hat{N}_j = \sum_k S_k \phi^S_{kj} + I_k \phi^I_{kj} \tag{4}$$

and

$$S_i + I_i = N_i. \tag{5}$$

Network topology can have a profound effect on the epidemic dynamics [7]. In general, different contact (interaction) topologies affect the critical thresholds R_0 [8, 9], as well as the shapes of epidemic curves [10]. However, meta-population models of the above type with a conserved mobility ($\sum_j \phi_{ij} = 1$), whilst producing distinct epidemic curves, have a critical threshold R_0 independent of the topology [6], due to spectral arguments. Intuitively this is due to mixing, and thus infection, occurring statistically within each meta population with constant β, as opposed to in proportion to the degree of the nodes in contact network models where, in effect, β is intrinsically tied to the local topology.

2 Maximum Entropy Method

The maximum entropy method is a statistical principle for choosing the functional form of an unknown probability distribution given a limited number of known properties or constraints [11, 12]. These constraints are typically normalization (it must be a probability distribution) in addition to defined averages of certain observables of that distribution. The method involves choosing the distribution that maximizes a functional of the probability distribution, namely the Shannon entropy, subject to these constraints. This distribution is considered to be the 'least-biased' distribution consistent with the known constraints.

The Shannon entropy of a random variable Y is defined as

$$H_Y = -\sum_y p_Y(y) \ln p_Y(y). \tag{6}$$

Normalization and other constraints are usually specified in the form

$$\sum_y p_Y(y) = 1 \tag{7}$$

$$\sum_y p_Y(x) f_i(y) = F_i,\ i \in \{1, \ldots, k\} \tag{8}$$

where F_i are constants equal to the mean of observable $f_i(y)$. Consequently the MaxEnt distribution $p_Y(y)$ is defined as

$$p_Y(y) = \left\{ \underset{q(y)}{\operatorname{argmax}} \ -\sum_y q(y) \ln q(y) \,\middle|\, \sum_y q(y) = 1 \ \wedge \ \sum_y q(y) f_i(y) = F_i \ \forall \ i \in \{1, \ldots, k\} \right\}. \tag{9}$$

This maximization is then achieved with the use of variational calculus and Lagrange multipliers.

The approach used in the main text is a slightly adapted form of the above methodology since we are interested in the least biased estimate of $\phi^x_{ij}(\mathbf{I}, \mathbf{C})$ which is a conditional probability, dependent on the compartment x and the origin location i. Consequently, the distribution over which we desire the maximum entropy is the following joint distribution

$$p(x, i, j) = p_X(x) p_L(i|x) p_M(j|i, x) \tag{10}$$

$$= p_X(x) p_L(i|x) \phi^x_{ij}(\mathbf{I}, \mathbf{C}) \tag{11}$$

which describes the probability of an individual being in compartment x ($x \in \{S, I\}$), being associated with location i ($i \in \{1, \ldots, m\}$), i.e. their home location, and mixing in location j ($j \in \{1, \ldots, m\}$) due to their mobility. This has been decomposed into a global probability of being in compartment X, $P_X(x)$, a probability of being associated with location i given that the individual is in compartment X, $P_L(i|x)$ and then finally the probability of mixing in location j due to mobility given that the individual is associated with location i and is in compartment X, $P_M(j|i, x)$. This final probability we recognize as the unknown mobility term $\phi^x_{ij}(\mathbf{I}, \mathbf{C})$. At any point in the evolution of the epidemic, the probabilities $P_X(x)$ and $P_L(i|x)$ are constants reflecting the state of the epidemic $\mathbf{I}$. Explicitly, for every pair $\{x, i\}$ we have a constant,

denoted $\kappa_{x,i}$ where

$$p_X(x) = \frac{\sum_i x_i}{\sum_x \sum_i x_i} \tag{12}$$

$$p_L(i|x) = \frac{x_i}{\sum_i x_i} \tag{13}$$

$$\sum_j p(x,i,j) = \frac{x_i}{\sum_x \sum_i x_i} = \kappa_{x,i}. \tag{14}$$

This specification is much stronger than the usual normalization constraint and leads to $M \times |\{S, I\}| = 2M$ constraints related to normalization given all combinations of (14).

In addition to this we specify certain additional constraints in terms of population averages. Over the total population we have a specified mean cost which is given by

$$\sum_{x,i,j} p(x,i,j) c_{ij} = C. \tag{15}$$

We then have two constraints on means over sub-populations for the compartments, namely the mean experienced benefit for infectious and susceptible individuals (B^I and B^S) such that

$$\sum_{i,j} p(I,i,j) b_j = p_X(I) B^I = \hat{B}^I \tag{16}$$

since here $p_X(I)$ is a constant. Similarly we have

$$\sum_{i,j} p(S,i,j) b_j = p_X(S) B^S = \hat{B}^S. \tag{17}$$

This maximization of the entropy of the distribution $p(x,i,j)$ subject to these constraints is given by

$$p(x,i,j) = \left\{ \underset{q(x,i,j)}{\text{argmax}} \; -\sum_x q(x,i,j) \ln q(x,i,j) \;\middle|\; \sum_j q(x,i,j) = \kappa_{x,i} \; \forall \, x,i \; \wedge \; \sum_{x,i,j} q(x,i,j) c_{ij} = C \right.$$
$$\left. \wedge \; \sum_{i,j} q(I,i,j) b_j = \hat{B}^I \; \wedge \; \sum_{i,j} q(S,i,j) b_j = \hat{B}^S \right\}. \tag{18}$$

We then find the MaxEnt solution by extremizing the following functional, where α^x, ω and $\eta_{x,i}$ are undetermined Lagrange multipliers,

$$\mathcal{J}[q] = \sum_x \left[\sum_{i,j} q(x,i,j) \ln q(x,i,j) - \alpha^x \left(\sum_{i,j} q(x,i,j) b_j - \hat{B}^x \right) \right] \tag{19}$$

$$- \sum_{x,i} \eta_{x,i} \left(\sum_j q(x,i,j) - \kappa_{x,i} \right) + \omega \left(\sum_{x,i,j} q(x,i,j) c_{ij} - C \right) \tag{20}$$

or equivalently

$$\mathcal{J}[q] = \sum_{x,i,j} q(x,i,j) \ln q(x,i,j) - \alpha^I \left(\sum_{x,i,j} \delta_{x,I} \, q(x,i,j) b_j - \hat{B}^I \right) - \alpha^S \left(\sum_{x,i,j} \delta_{x,S} \, q(x,i,j) b_j - \hat{B}^S \right) \tag{21}$$

$$- \sum_{x,i} \eta_{x,i} \left(\sum_j q(x,i,j) - \kappa_{x,i} \right) + \omega \left(\sum_{x,i,j} q(x,i,j) c_{ij} - C \right), \tag{22}$$

where $\delta_{x,y}$ is the Kronecker delta. We note that the sign of the Lagrange multipliers is generally one of convention, however we specify the above since we interpret B^x as a benefit (which increases probabilities) and C as a cost (which decreases probabilities). We proceed by computing the functional derivative which is given by

$$\frac{\delta J}{\delta q} = 1 + \ln q(x,i,j) - \sum_{x,i} \kappa_{x,i} - \delta_{x,I}\alpha^I b_j - \delta_{x,S}\alpha^S b_j + \omega c_{ij}. \tag{23}$$

The MaxEnt solution is given by considering the derivative to vanish such that

$$p(x,i,j) = \exp\left[-1 + \sum_{x,i} \kappa_{x,i} + \delta_{x,I}\alpha^I b_j + \delta_{x,S}\alpha^S b_j - \omega c_{ij}\right] \tag{24}$$

$$= \hat{Z}_{x,i}^{-1} \exp\left[\delta_{x,I}\alpha^I b_j + \delta_{x,S}\alpha^S b_j - \omega c_{ij}\right] \tag{25}$$

with the last line emerging from grouping all constant terms into a single pre-factor, or partition sum. Finally, we recognize that $p(x,i,j) \propto \phi_{ij}^x(\mathbf{I},\mathbf{C})$ differing only by specified constants ($\kappa_{x,i}$) which can be incorporated into the partition sum such that we finally have

$$\phi_{ij}^x(\mathbf{I},\mathbf{C}) = Z_{x,i}^{-1} \exp[\alpha^x b_j - \omega c_{ij}] \tag{26}$$

with

$$Z_{x,i} = \sum_j \exp[\alpha^x b_j - \omega c_{ij}] \tag{27}$$

ensuring normalization.

Consequently the functional form of the behaviour of infectious individuals is entirely captured by the two Lagrange multipliers α^I and ω whilst the behaviour of susceptible individuals is captured by α^S and ω. There is a duality between these parameters and the mean observables B^I, B^S and C. Consequently this methodology is very flexible. One could in principle measure such observables from data and thus deduce the most likely Lagrange multipliers for a minimal empirical model or specify the Lagrange multipliers as behavioural parameters which lead to different mean observables, based on a minimal behavioural model. The latter is what is performed in the main text.

Finally, we point out that many of the above choices surrounding the number of constraints can be relaxed in order to specify as highly a parametrized model as desired. For instance, in precisely the same way that we implemented a benefit constraint for each compartment (B^I, B^S), one could implement a cost constraint for each compartment also. Similarly one could utilize a separate constraint for each location i such that there would be a distinct Lagrange multiplier related to benefit and cost (α^x, ω) for each location. This would allow for topologically inhomogenous behaviours. We note, however, that this is always at the cost of bringing more parameters into one's model at the expense of parsimony, overfitting and/or lack of relevant data.

References

[1] Roy M Anderson and Robert M May. *Infectious diseases of humans: Dynamics and control.* Oxford university press, 1992.

[2] William Ogilvy Kermack and Anderson G McKendrick. A contribution to the mathematical theory of epidemics. *Proceedings of the Royal Society of London. Series A, Containing papers of a mathematical and physical character*, 115(772):700–721, 1927.

[3] Odo Diekmann, Johan Andre Peter Heesterbeek, and Johan AJ Metz. On the definition and the computation of the basic reproduction ratio R0 in models for infectious diseases in heterogeneous populations. *Journal of Mathematical Biology*, 28(4):365–382, 1990.

[4] James O Lloyd-Smith, Sebastian J Schreiber, P Ekkehard Kopp, and Wayne M Getz. Superspreading and the effect of individual variation on disease emergence. *Nature*, 438(7066):355, 2005.

[5] Lucas M Stolerman, Daniel Coombs, and Stefanella Boatto. Sir-network model and its application to dengue fever. *SIAM Journal on Applied Mathematics*, 75(6):2581–2609, 2015.

[6] Julien Arino and P Van den Driessche. A multi-city epidemic model. *Mathematical Population Studies*, 10(3):175–193, 2003.

[7] Matt Keeling. The implications of network structure for epidemic dynamics. *Theoretical Population Biology*, 67(1):1–8, 2005.

[8] Romualdo Pastor-Satorras and Alessandro Vespignani. Epidemic spreading in scale-free networks. *Physical Review Letters*, 86(14):3200, 2001.

[9] Romualdo Pastor-Satorras and Alessandro Vespignani. Epidemic dynamics and endemic states in complex networks. *Physical Review E*, 63(6):066117, 2001.

[10] Matt J Keeling and Ken TD Eames. Networks and epidemic models. *Journal of the Royal Society Interface*, 2(4):295–307, 2005.

[11] Edwin T Jaynes. Information theory and statistical mechanics. *Physical Review*, 106(4):620, 1957.

[12] Edwin T Jaynes. Information theory and statistical mechanics. ii. *Physical Review*, 108(2):171, 1957.
